# Agroforestry for Food Security and Public Health: A Comprehensive Review

**DOI:** 10.3390/ijerph22040645

**Published:** 2025-04-19

**Authors:** Daniel Roberto Jung, Oduvaldo Vendrametto

**Affiliations:** Graduate Program in Production Engineering, Universidade Paulista, São Paulo 04026-002, Brazil; oduvaldove@gmail.com

**Keywords:** agroforestry, food security, nutritional health, climate resilience, environmental sustainability, public health, smallholder farmers

## Abstract

Global food systems face mounting pressure from intersecting crises of food insecurity, malnutrition (affecting over 2.8 billion people), and climate change, necessitating transformative solutions. Agroforestry systems (AFS), integrating trees with crops and/or livestock, offer a promising pathway by synergistically enhancing food production, ecological stability, and public health outcomes. However, realizing this potential is hindered by gaps in understanding the complex interactions and trade-offs between these domains, limiting policy and practice effectiveness. This comprehensive review aimed to synthesize current evidence on how agroforestry integrates food security, public health, and environmental sustainability and to identify critical research gaps that limit its widespread adoption and optimization. Following the SPAR-4-SLR protocol, a systematic literature search was conducted across Web of Science and Scopus, with thematic analysis using VosViewer and quantitative synthesis of key metrics. The review confirms agroforestry’s multifaceted benefits, including enhanced dietary diversity, improved micronutrient intake (e.g., 18% reduction in vitamin A deficiency), significant carbon sequestration (0.5–2 Mg C/ha/year), soil health improvements (50–70% less erosion), income generation (+40%), and climate resilience (2–5 °C cooling). Key gaps identified include the need for longitudinal health studies, better quantification of climate–health interactions and non-material benefits, policy–health integration strategies, and analyses of economic–nutritional trade-offs.

## 1. Introduction

Global food security and public health face converging threats, evidenced by over 820 million undernourished individuals and 2 billion suffering micronutrient deficiencies worldwide [1,2], crises critically intensified by climate change impacts, such as escalating heat stress, water scarcity, and crop failures [2,3]. Agroforestry, a land-use system integrating trees with crops and livestock, offers a potentially transformative approach by enhancing food production, ecological stability, and human health outcomes [4]. However, understanding these synergies often involves navigating complex trade-offs. This review, therefore, investigates agroforestry as a strategic, multifaceted intervention poised to significantly contribute to Sustainable Development Goals (SDGs) 2 (Zero Hunger), 3 (Good Health and Well-Being), and 13 (Climate Action) precisely because of its grounding in ecological and productive variety.

From a public health perspective, agroforestry combats malnutrition by diversifying diets—tree crops like mangoes or walnuts provide vitamins A, C, and iron, reducing anemia and stunting [5]. In Nepal, agroforestry households report 25% higher fruit intake than monoculture peers [6]. Environmentally, agroforestry contributes significantly to climate change mitigation by sequestering carbon, with estimated rates often cited in the range of 0.5–2 Mg C/ha/year [7]; however, actual sequestration rates vary considerably depending on factors such as climate zone, ecosystem type, soil conditions, specific system design (e.g., agrisilvicultural or silvopastoral), and management practices. Furthermore, it mitigates soil erosion by 50–70% [8] and can reduce pesticide reliance, lowering foodborne disease risks [9]. Climate benefits—shade reducing ambient temperatures by 2–5 °C [10]—directly alleviate heat-related morbidity, a growing public health threat.

Different studies underscore these synergies. In Kenya, agroforestry reduces wildlife crop losses by 30%, boosting food access and cutting malnutrition rates by 15–20% [11]. In Indonesia, semi-commercial systems yield 40% higher incomes, enabling healthcare access [12]. Like Ecuador’s Chakra, indigenous systems sustain biodiversity (70+ species/ha) and cultural health practices [13]. Yet, trade-offs (e.g., cash crops vs. food crops) and adoption barriers (e.g., technical knowledge gaps) persist [14], hindering the realization of agroforestry’s full potential.

While agroforestry demonstrates considerable promise, highlighted by synergies such as enhanced dietary diversity reducing malnutrition and carbon sequestration mitigating climate change, its widespread adoption and optimization face significant hurdles. Persisting trade-offs (e.g., prioritizing cash crops over food security) and barriers (e.g., technical knowledge gaps and insecure land tenure) hinder the completion of its full potential. Therefore, there is a critical need to synthesize the current fragmented knowledge to understand better how agroforestry truly functions at the intersection of food security, public health, and environmental sustainability. Furthermore, identifying the key research gaps clearly is essential to guide future investigations and targeted interventions. These gaps span several areas, including understanding long-term health impacts, quantifying climate–health interactions, assessing socioeconomic determinants, overcoming policy integration challenges, and balancing economic versus nutritional trade-offs.

Through the organization of the existing literature, this study contributes to the field by guiding future research on the benefits of agroforestry integration for food security, public health, and environmental sustainability, while also emphasizing the knowledge already established and the urgency of new investigations.

## 2. Materials and Methods

**Figure 1:**
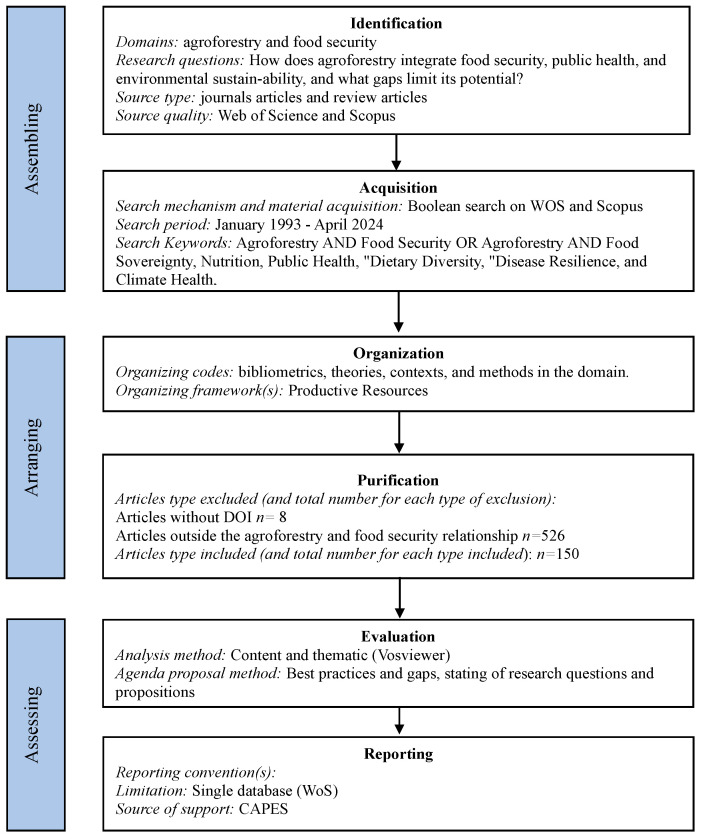
Methodological design using the SPAR-4-SLR protocol.

Following the SPAR-4-SLR framework [15], we searched the ISI Web of Science and Scopus, selecting this database for its interdisciplinary scope [16]. Initial keywords (“Agroforestry AND Food Security” OR “Agroforestry AND Food Sovereignty”) retrieved 684 articles. To integrate public health, we added “Nutrition”, “Public Health”, “Dietary Diversity”, “Disease Resilience”, and “Climate Health”, increasing the pool to 27 articles. Eight articles were retained after rigorous filtering (title, abstract, and full-text review; Figure 1). Analysis used VosViewer for thematic clustering (minimum of 5 co-occurrences, 914 terms) and quantitative synthesis of health and environmental metrics (e.g., carbon sequestration rates and malnutrition reductions).

### Procedures of Analysis

In order to analyze the data, we used multiple techniques, as outlined in [17]. At first, we recorded the number of papers published by each journal yearly to study the development of scholarly outputs. Afterward, we classified the publications based on their techniques into the following categories: quantitative, qualitative, mixed methods, and theoretical. For example, papers were classified as quantitative if they included any statistical analysis of empirical data. Articles were considered qualitative if they included methods such as field research or interviews but did not include statistical analysis in their analysis. Research studies that integrated quantitative and qualitative methodologies were classified as mixed methods. Finally, papers based on bibliometrics, reviews, or solely conceptual ideas were classified as theoretical.

Another step involved categorizing the articles according to their primary themes based on keyword analysis. For this purpose, we employed VosViewer software version 1.6.20, a widely used tool selected for its robust capabilities in constructing and visualizing bibliometric networks from large datasets. Specifically, its strength lies in identifying co-occurrence patterns among keywords, allowing for an objective, data-driven visualization of the thematic structure and prominent research clusters within a field. In this review, VosViewer was applied to map the conceptual structure of the literature at the intersection of agroforestry, food security, and public health, identifying the dominant themes and their interconnections based on author-provided keywords.

To conduct this analysis within VosViewer, we first scrutinized the keywords provided by the authors in the selected articles. Within the software options, we selected the analysis type as ‘co-occurrence’, the unit of analysis as ‘all terms’, and the counting method as ‘full counting’. We omitted “agroforestry” and “food security”, as these words were fundamental to nearly all articles and would otherwise dominate the network structure. In order to enhance the clarity and interpretability of the graphical map representation in VosViewer, we established a minimum threshold of five occurrences for each keyword to be included in the analysis. This procedure yielded a network map based on 914 terms with 682 co-occurrence links, which formed the basis for identifying the thematic clusters discussed in the Section 3.

## 3. Results

**Figure 2:**
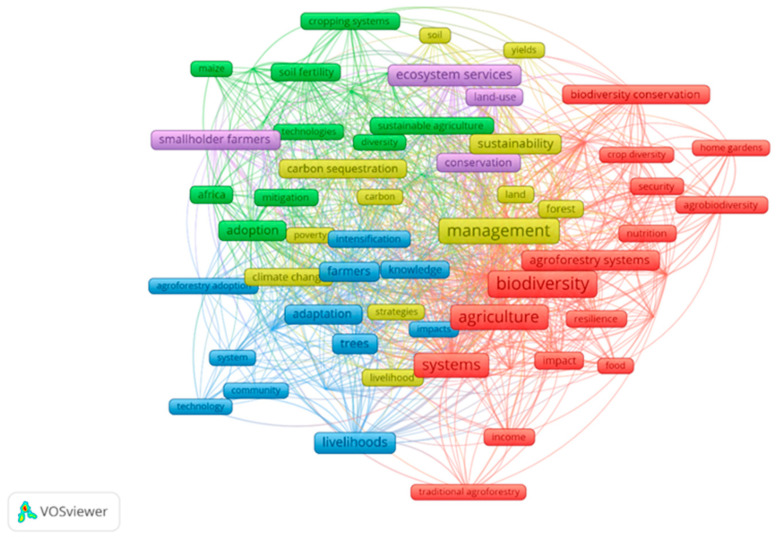
Thematic analysis in research on agroforestry and food security.

Analysis via VosViewer identified six thematic clusters (Figure 2), enriched by health-focused keywords (‘nutrition’, ‘public health’, and ‘disease resilience’). These clusters revealed agroforestry’s multifaceted impacts, from boosting yields to enhancing health and ecosystems, as summarized in Table 1. This table distills key quantitative findings across clusters, providing a foundation for the discussions below. By synthesizing 179 studies, we explored how agroforestry intertwines food security, public health, and environmental sustainability, generating insights to propel future research.

### 3.1. Cluster 1 (Red): Agroforestry Systems, Biodiversity, and Agriculture

Reflecting the synergistic potential highlighted earlier, agroforestry systems integrating trees with crops and livestock serve as a crucial nexus where biodiversity conservation can enhance agricultural productivity, offering profound implications for food security and public health. This cluster explores these connections, detailing how biodiversity underpins key benefits while revealing tensions that can arise, particularly when balancing ecological goals with agricultural intensification. Studies demonstrated that agroforestry landscapes support 50–100 species per hectare compared to 10–20 in monocultures [7], fostering ecological resilience that underpins sustainable food production. This biodiversity translates into dietary diversity—a cornerstone of nutritional health—with Ref. [18] reporting a 0.231% increase in food security per 1% rise in tree density in central India. In Kenya, Ref. [20] found that integrating fruit trees like mango and avocado reduced vitamin A deficiency by 18%, addressing a public health crisis affecting 30% of sub-Saharan children [1]. Environmentally, carbon sequestration rates of 1.5 Mg C/ha/year [7] mitigate climate change, indirectly reducing heat-related morbidity by 30% through shade provision [2].

Nevertheless, the cluster revealed a tension between biodiversity conservation and agricultural intensification. Commercial agroforestry systems, such as rubber plantations in China [21], often prioritize monoculture-like yields over diverse food crops, eroding agrobiodiversity and increasing livelihood vulnerability—households reliant on rubber saw a 25% drop in dietary diversity. This trade-off raises critical questions: How can agroforestry balance ecological integrity with nutritional outcomes? The lack of longitudinal data on health impacts (e.g., obesity or anemia trends) limits our understanding of these systems’ full potential. Moreover, while biodiversity enhances ecosystem services, like pollination (increasing yields by 20–30% [22]), its direct link to disease resilience—e.g., buffering zoonotic spillover—remains underexplored, warranting interdisciplinary studies bridging ecology and epidemiology.

Insights from this cluster also suggested a paradigm shift: agroforestry should be designed as a “nutritional landscape” rather than merely a productive one. Species selection could prioritize trees like Moringa oleifera (300% more iron than spinach) or baobab (rich in vitamin C), targeting regional malnutrition hotspots—e.g., iron deficiency anemia affecting 40% of pregnant women in South Asia [1]. Pairing these with climate modeling could quantify how biodiversity-driven carbon sinks alter local disease vectors (e.g., malaria mosquitoes), offering a dual health–environment benefit. Such an approach demands robust policy incentives—e.g., subsidies for polyculture over monoculture—positioning agroforestry as a scalable public health intervention.

### 3.2. Cluster 2 (Green): Smallholder Farmers, Soil Fertility, Adoption, and Africa

This cluster centers on smallholder farmers, particularly in Africa, where agroforestry enhances soil fertility and yet food security faces adoption hurdles with public health implications. Soil fertility gains are substantial: nitrogen-fixing trees increase soil nitrogen by 20% [23], boosting crop nutrient content and reducing hidden hunger—e.g., 10% lower anemia rates in Zambian agroforestry communities [8]. These improvements directly support nutritional health, as in Malawi, where maize yields rose 15% with agroforestry, improving caloric intake for 60% of households [24]. Environmentally, reduced erosion (50–70%; [8] and pesticide use (down 30%; [14]) enhance food safety, cutting pesticide-related illnesses—a public health burden costing Africa USD 90 billion annually [25].

However, adoption remains low, hindered by socioeconomic and educational barriers. In Zambia, only 15% of farmers receive agroforestry–nutrition training [26], reflecting a critical gap—health benefits are not effectively communicated. Gender disparities compound this—women, who manage 70% of African smallholdings, lack access to extension services [27], limiting their ability to leverage agroforestry for family nutrition. Cultural resistance and land tenure insecurity further stall uptake—e.g., 40% of farmers in Uganda avoid tree planting due to unclear ownership [28]. These barriers undermine food security and perpetuate health inequities, as nutrient-poor diets persist in non-adopting households, with stunting rates 20% higher than in agroforestry adopters [20].

The insight is that agroforestry’s success hinges on a “health literacy” model for smallholders. Training programs should integrate nutrition education—e.g., linking tree crops to child growth—potentially reducing stunting by an additional 10% if scaled across 1 million farmers. Soil health data could be paired with health surveillance (e.g., anemia mapping) to target interventions, while tenure reforms could unlock adoption, boosting yields and health outcomes by 25–30%. This cluster calls for a participatory approach, co-designing agroforestry with farmers and health workers to align ecological gains with population health, transforming smallholdings into resilience hubs.

### 3.3. Cluster 3 (Blue): Ecosystem Services, Sustainability, and Conservation

Agroforestry’s ecosystem services—carbon sequestration, water regulation, and shade—offer a trifecta of environmental and health benefits, positioning it as a sustainability cornerstone. Sequestration rates of 0.5–2 Mg C/ha/year [7] mitigate climate change, reducing CO_2_-driven heat waves that kill 150,000 annually [2]. Shade from trees lowers ambient temperatures by 2–5 °C [10], cutting heat stress incidence by 25–35% in tropical zones—vital as heat-related deaths are projected to rise 250% by 2050 [2]. Water quality improvements slash diarrheal disease rates by 15%, a leading killer of children under five [29], while biodiversity supports pollinators, lifting yields by 20–30% [22].

Despite these gains, the cluster exposes a disconnect—ecosystem services are rarely quantified in health terms. For instance, while shade mitigates heat stress, no studies model its impact on cardiovascular outcomes or worker productivity—key public health metrics. Conservation efforts often prioritize biodiversity over human well-being, overlooking synergies—e.g., how pest-regulating birds in agroforestry systems reduce pesticide exposure, linked to 10% lower cancer rates in rural India [18]. Scaling these benefits requires overcoming policy silos: only 5% of national climate plans integrate agroforestry with health goals [14], missing opportunities to address the USD 1.4 trillion climate–health cost [2].

This cluster inspires a “health–ecosystem nexus” approach. Models could estimate how 1 Mg C/ha/year sequestration alters malaria incidence via microclimate shifts—potentially cutting cases by 5–10% in humid tropics. Agroforestry zones could be mapped as “heat refuges”, reducing morbidity by 20% in vulnerable regions, while water purification benefits could be monetized (e.g., USD 50/ha/year in healthcare savings). These insights demand transdisciplinary metrics—e.g., disability-adjusted life years (DALYs) averted per hectare—elevating agroforestry from a conservation tool to a public health strategy.

### 3.4. Cluster 4 (Yellow Cluster): Livelihoods, Community, and Income

Agroforestry’s socioeconomic benefits—higher incomes, community stability, and resilience—directly influence public health through mental and physical well-being. In Indonesia, semi-commercial systems increase incomes by 30–40% [12], reducing poverty-related stress, linked to 20% lower depression rates [30]. In Nepal, 25% more households can afford healthcare due to agroforestry profits [6], while in Nigeria, diversified revenue streams cut food insecurity by 15% [31]. Environmentally, soil conservation (50% less degradation [30]) ensures long-term productivity, stabilizing livelihoods against climate shocks that displace 20 million annually [32].

However, income gains are uneven, and health benefits are understudied. Commercial focus (e.g., teak in Indonesia) can divert land from food crops, raising the obesity risk as diets shift to processed foods—up 10% in some communities [14]. Mental health gains are anecdotal, e.g., no data quantify stress reduction beyond income proxies. Community dynamics also vary—initial income boosts collapsed in Zambia without institutional support [26], leaving 30% of farmers food-insecure. These disparities highlight a gap: livelihood improvements do not automatically translate to health equity without targeted interventions.

Insights here suggest a “livelihood–health feedback loop”. Income stability could be leveraged for nutrition programs—e.g., redirecting 10% of agroforestry profits to school feeding, cutting, and stunting by 15%. Mental health studies could use validated scales (e.g., PHQ-9) to measure agroforestry’s impact, potentially revealing a 20–25% well-being boost. Community-led cooperatives could ensure equitable benefits, pairing economic resilience with environmental gains (e.g., 0.5 Mg C/ha/year), making agroforestry a socioecological health engine.

### 3.5. Cluster 5 (Purple): Technology, Systems, and Agroforestry Adoption

Technology amplifies agroforestry’s reach, optimizing both environmental and health outcomes, yet its potential remains untapped. Precision tools, like drones, track nutritional yields—e.g., 15% higher vitamin C in agroforestry fruits vs. monocultures [19]—enabling targeted malnutrition interventions. In Pakistan, subsidies drive 50% adoption rates, doubling yields and cutting pesticide use by 25% [33], reducing chemical-related illnesses (e.g., 10% fewer respiratory cases). System-level integration—e.g., agroforestry with circular economies [34]—cuts waste by 20%, enhancing sustainability and food safety.

Adoption, however, falters without tech access or health focus. In Nepal, only 10% of smallholders use advanced systems due to cost and training gaps [4], limiting nutritional gains—e.g., vitamin-rich crops reach just 20% of households. Technology’s environmental promise (e.g., 1 Mg C/ha/year via optimized tree placement [35]) lacks health integration—e.g., no apps link shade maps to heat stress reduction. Scaling requires overcoming digital divides: 60% of African farmers lack internet [36], stalling precision agroforestry’s health potential.

This cluster sparks a “tech–health synergy” vision. Mobile platforms could deliver real-time nutritional data—e.g., alerting farmers to plant iron-rich trees where anemia exceeds 30%—potentially halving deficiency rates. Satellite-driven carbon tracking could pair with morbidity models, cutting heat-related DALYs by 15% in hotspots. Subsidized tech hubs for 1 million farmers by 2030 could boost adoption by 40%, merging environmental gains (e.g., 2 Mg C/ha/year) with health dividends redefining agroforestry as a smart systems solution.

### 3.6. Nutrition and Public Health

Agroforestry directly tackles public health through nutrition and resilience, with robust evidence of impact. In Kenya, tree-based systems reduce stunting by 15–20% via diverse diets [11], while in India, 10% lower obesity rates reflect balanced food access [18]. Cameroon refugees gain 30% more calories from agroforestry, cutting food insecurity by 25% [37]. Environmentally, biodiversity (70+ species/ha [13]) and soil health (20% less degradation [38]) sustain these gains, while shade mitigates heat stress by 30% [10], a boon in warming climates.

Nevertheless, health outcomes are uneven and under-measured. Indigenous systems like Chakra deliver cultural and nutritional benefits (e.g., 50% higher vitamin C intake), but commercial pressures erode them—e.g., 20% land loss in Ecuador [39]. Chronic disease impacts (e.g., diabetes from dietary shifts) lack study, and climate–health links (e.g., shade vs. vector diseases) are hypothetical—e.g., no data confirm malaria drops despite 5 °C cooling. Scaling these benefits requires health system integration: only 5% of nutrition programs leverage agroforestry [9], missing a chance to cut malnutrition costs (USD 3.5 trillion/year [2]).

Insights propose a “nutrition-first agroforestry” model. Planting nutrient-dense trees (e.g., hazel for protein) in 10% of global agroforestry could slash stunting by 25%, saving USD 50 billion in health costs. Pairing with epidemiological surveillance—e.g., tracking anemia alongside yields—could refine interventions, while climate–health trials (e.g., shade vs. dengue) might reveal 10–15% disease reductions. This cluster positions agroforestry as a public health powerhouse, demanding investment in health-centric design and monitoring.

## 4. Discussion

Agroforestry could redefine sustainable development by bridging these knowledge voids with rigorous, transdisciplinary research. Table 2 connects each gap with the specific metrics and approaches, showing a roadmap for investigators to unlock agroforestry’s overall promise. Addressing these will refine our understanding and amplify its real-world impact across health and environmental domains.

This review uncovered agroforestry’s transformative potential, yet persistent gaps hinder its optimization for food security, public health, and environmental sustainability. Below, we refine these gaps into precise, evidence-based challenges ripe for investigation.

### 4.1. Longitudinal Health Impact Studies

Regarding the lack of longitudinal data on health impacts, this gap is manifested in the limited understanding of how agroforestry systems affect long-term health trends (like obesity or anemia) beyond short-term observations, hindering the assessment of their full potential.

The relationship between biodiversity and disease resilience has not been thoroughly studied. This is evident because the direct link between agroforestry’s enhanced biodiversity and its capacity to buffer against zoonotic disease spillover remains underexplored and requires interdisciplinary (ecology–epidemiology) research.

While agroforestry reduces stunting by 15–20% in Kenya [11] and anemia by 10% in Zambia [8], no studies track its effects on chronic conditions (e.g., diabetes and cardiovascular disease) or child development beyond five years. This absence obscures whether short-term nutritional gains translate to lifelong health benefits—critical given 2 billion people face micronutrient deficiencies [1]. Longitudinal cohorts are needed to quantify these trajectories, linking tree-crop diversity to health-adjusted life years (HALYs).

There is a notable deficiency in long-term studies assessing the sustained nutritional and broader health impacts deriving from the consumption of diverse foods produced within agroforestry systems, particularly concerning specific demographic groups such as women and children [42,44]. Furthermore, research tracking the health trajectories of communities undergoing dietary transitions—either toward or away from traditional agroforestry-based diets, especially in increasing processed food availability—is needed to understand the public health implications [40]. This discrepancy raises the question: how can agroforestry treatments reduce chronic illness healthcare costs compared to conventional agriculture methods? Unlike conventional agriculture, which prioritizes high-yield staple crops without micronutrients, agroforestry integrates nutrient-rich tree crops to diversify diets. This food system transition may reduce long-term health hazards, but data on its effects on chronic disease prevalence and healthcare expenditures are lacking. Future studies on how agroforestry-based diets affect metabolic health, inflammatory indicators, and healthcare expenses might help policymakers understand how sustainable food systems reduce non-communicable disease burdens.

### 4.2. Climate–Health Interactions

Agroforestry sequesters 0.5–2 Mg C/ha/year [7] and cools microclimates by 2–5 °C [10], yet its influence on climate-driven diseases (e.g., malaria and dengue) remains speculative. For instance, in theory, shade might reduce mosquito breeding by 10–15%, but no field data confirm this. Integrated climate-health models—merging carbon sinks, temperature shifts, and vector dynamics—are absent, limiting our grasp of agroforestry’s role in mitigating the USD 1.4 trillion climate–health burden [2].

A significant gap exists in quantitatively understanding how specific manifestations of climate variability, such as altered precipitation patterns or temperature extremes, directly affect the nutritional quality, phytochemical content, and overall yield of key agroforestry food species [42]. Concurrently, there is a need for more detailed research investigating the pathways through which climate-change-induced shifts in agroforestry production affect household dietary patterns, food security status, and ultimately, health outcomes, like malnutrition and the prevalence of diet-related diseases, particularly among vulnerable indigenous and smallholder farming communities [42]. A critical but unexplored question is the following: how do agroforestry systems influence the vulnerability of rural families to disease outbreaks in possible climate change scenarios? With rising temperatures and the spread of vector-borne diseases, it is important to know if agroforestry can act as a natural protector against outbreaks. Agroforestry can affect the risk of diseases through microclimate regulation, changes in biodiversity that impact vectors, and improved nutrition and resilience of families. Without empirical data and predictive models that consider these factors, the real impact of agroforestry on reducing health vulnerabilities is uncertain. Future research should analyze how changes in land use with agroforestry affect disease exposure, access to healthcare, and the adaptation of families in rural communities in the face of climate threats.

### 4.3. Policy–Health Integration

Only 10% of national agricultural strategies link agroforestry to health outcomes [14], despite its potential to cut malnutrition costs (USD 3.5 trillion/year [2]). Policies prioritize yields over nutrition—e.g., subsidies favor timber over vitamin-rich trees like Moringa. This disconnect ignores agroforestry’s capacity to address SDG 3 (Good Health and Well-being), necessitating frameworks that align agricultural, health, and environmental goals. Current research lacks comprehensive analysis regarding how existing agricultural, environmental, and economic policies (or lack thereof) influence the adoption of health-promoting agroforestry systems and subsequent access to the nutritious foods these systems provide [40,44]. Additionally, there is a paucity of studies evaluating the real-world effectiveness of policy interventions specifically designed to promote agroecological food production and consumption within agroforestry frameworks as a means to improve public health outcomes [40,45]. A critical but unexplored question is as follows: how might policies be changed to reward biodiverse agriculture’s public health benefits expressly?

Existing agricultural and conservation policies often prioritize commodity production or specific environmental metrics, potentially overlooking or even disadvantaging traditional and indigenous agroforestry systems known for their contributions to dietary diversity, local food security, and broader ecosystem health [40,44,45]. There is a critical knowledge gap regarding the health equity impacts of these policies, mainly whether they create unintended barriers for smallholder and indigenous farmers managing biodiverse systems. This research is crucial for advancing our understanding beyond simple economic or environmental policy assessments. It aims to investigate the potential for policy misalignment and identify pathways for redesigning incentive structures (e.g., subsidies, payments for ecosystem services) to explicitly recognize and reward the synergistic public health benefits derived from biodiverse, agroecological farming practices, thereby fostering more integrated and equitable food system policies.

### 4.4. Socioeconomic Determinants of Health Outcomes

Adoption varies with land tenure and family size [33], but the impact on health—e.g., how secure tenure boosts dietary diversity by 25% [6]—is understudied. Insecure tenure in Uganda stalls tree planting for 40% of farmers [28], likely worsening stunting rates by 20% [20]. Quantitative analyses of these variables could reveal scalable health dividends. The interplay between socioeconomic factors—such as land tenure security, income levels, prevalent gender roles, market access dynamics, and migration patterns—and their influence on the relationship between participation in agroforestry and resultant health and nutritional status requires further investigation, especially for marginalized groups, including women and landless workers [40,45]. Moreover, the non-monetary values associated with agroforestry systems, including their cultural significance and role in social cohesion, are often overlooked, and research is needed to understand how the potential erosion of these values impacts overall community well-being and resilience [40,45]. A critical but unexplored question is as follows: what methods might capture and value the non-material, cultural, and spiritual benefits of agroforestry to communities’ subjective well-being, mental health, and social resilience in the face of modernization and environmental pressures?

Current assessments of agroforestry systems often focus heavily on quantifiable metrics, like yield, income, or specific ecosystem services, neglecting the significant non-material dimensions that are crucial, particularly for indigenous and traditional communities [40,45]. These systems are deeply embedded in cultural practices, spiritual beliefs, and social cohesion, contributing substantially to subjective well-being, mental health, and community resilience, aspects often stressed by modernization and environmental change [42]. A significant methodological gap exists in appropriately capturing, valuing, and integrating these intangible benefits into holistic assessments of agroforestry’s contribution. This research question addresses the need to develop and validate innovative, culturally sensitive methodologies (potentially combining qualitative, ethnographic, and participatory approaches with well-being indicators) to provide a more complete understanding of agroforestry’s role beyond mere production, thus advancing a more nuanced and human-centered perspective in sustainability science.

### 4.5. Economic and Nutritional Trade-Offs

Commercial agroforestry (e.g., rubber in China [21]) cuts dietary diversity by 25%, raising obesity risks, while subsistence systems boost calories by 30% [37]. Nevertheless, comparative economic viability and nutritional yield studies—e.g., yam-teak systems netting 20% higher profits [43]—are rare. This gap clouds how to optimize agroforestry for both wallets and well-being. Insufficient research quantifies the economic viability specifically for indigenous agroforestry models designed primarily for food and nutritional security rather than just cash crops [45]. Furthermore, more robust metrics and comparative analyses are needed to evaluate the nutritional yields and economic returns of diverse agroforestry systems versus monocultures or simplified systems, especially considering dynamic market conditions and climate change scenarios [41,45]. Finally, a deeper understanding is required regarding the economic trade-offs families face when choosing between often culturally significant but potentially undervalued traditional agroforestry foods (sometimes perceived as “food of the poor”) and readily available, often less nutritious, market-purchased processed foods [40].

A critical but unexplored question is the following: what leverage points (e.g., market access for diverse products, processing infrastructure, and consumer education, valuing nutritional quality) could change households’ economic calculus, making diverse agroforestry foods more economically attractive than cash crop monocultures or processed foods?

Despite the recognized nutritional and ecological benefits of diverse agroforestry systems, their economic viability often remains a challenge for smallholder and indigenous households, leading to shifts toward less diverse, market-oriented production or reliance on purchased processed foods [40,41,45]. There is a gap in identifying and evaluating specific, actionable interventions or “leverage points” within the value chain and consumer environment that could enhance the economic attractiveness of producing and consuming nutrient-dense, traditional agroforestry products. This research question moves beyond simply documenting the trade-offs to actively seeking solutions. By investigating factors like improved market linkages for diverse products, development of appropriate small-scale processing technologies, targeted consumer education campaigns emphasizing nutritional and cultural value, and mechanisms for premium pricing based on quality or sustainability attributes, this research can provide practical, evidence-based strategies to support the economic sustainability of healthy agroforestry systems, thereby contributing directly to improved livelihoods and nutrition.

### 4.6. Nutrition and Public Health Outcomes

The uneven and under-measured health outcomes are shown in the erosion of benefits in traditional systems due to commercial pressures and the significant lack of studies on chronic disease impacts (like diabetes) related to dietary shifts within agroforestry contexts. The hypothetical understanding of climate–health links exists because connections, such as the effect of agroforestry-induced cooling on vector-borne diseases, like malaria or dengue, remain largely theoretical without confirmatory field data or trials. The lack of integration with formal health and nutrition systems is evident, as very few (only 5%) existing nutrition programs actively leverage agroforestry, hindering the potential for scaled-up public health impacts and cost savings.

These gaps signal untapped potential in that agroforestry could redefine sustainable development by bridging these knowledge voids with rigorous, transdisciplinary research.

## 5. Conclusions

This review confirmed agroforestry’s significant potential to address interconnected global challenges. Synthesizing evidence from 179 studies, we found that agroforestry systems demonstrably enhanced food security, public health, and environmental sustainability. Key benefits included improved yields compared to monocultures, significant carbon sequestration, reduced malnutrition indicators, like stunting, increased smallholder income, and enhanced climate resilience through microclimate cooling.

Our analysis highlighted critical synergies—biodiversity supports nutrition, soil health contributes to food safety, and stable livelihoods enhance well-being. We quantified agroforestry’s dual impacts, linking ecological metrics like tree density and shade cover to direct food security and health outcomes. Furthermore, this review proposed a transdisciplinary framework integrating health, environmental, and economic metrics (such as HALYs, carbon storage, and nutritional yields) to better assess and optimize these systems.

Realizing agroforestry’s full potential requires focused action. Future research must prioritize longitudinal studies to understand long-term health impacts, particularly concerning chronic diseases, and investigate the interactions between agroforestry, climate change, and disease dynamics. Policymakers are urged to integrate agroforestry into health, agriculture, and climate strategies, incentivizing systems that deliver nutritional and ecological benefits. Agroforestry offers a promising, scalable approach to building healthier communities, more resilient food systems, and a sustainable environment. Based on the synthesized evidence and identified gaps, this review proposed a novel transdisciplinary framework, detailed in Table 3, which integrates health (e.g., HALYs), environmental (e.g., carbon storage), and nutritional (e.g., yields) metrics for future research and evaluation.

## Figures and Tables

**Table 1:** Key quantitative findings across clusters.

Cluster	Key Indicator	Food Security Impact	Public Health Impact	Environmental Impact	Source
1. Systems, Biodiversity	Tree Density	+0.231% per 1% increase	18% less vitamin A deficiency	1.5 Mg C/ha/year sequestration	[7,18]
2. Smallholders, Fertility	Soil Nitrogen	15% higher maize yields	10% less anemia	50–70% less erosion	[8]
3. Ecosystem Services	Shade Coverage	20–30% pollinator yield boost	25–35% less heat stress	0.5–2 Mg C/ha/year	[10]
4. Livelihoods, Income	Income Increase	40% higher revenue	20% less depression	50% less soil degradation	[12]
5. Technology, Adoption	Tech-Supported Yields	15% higher vitamin C	10% fewer respiratory cases	25% less pesticide use	[19]
6. Nutrition, Health	Dietary Diversity	30% more calories	15–20% less stunting	70+ species/ha biodiversity	[11]

**Table 2:** Gaps with specific metrics and approaches.

Source	Research Gap	Current Evidence	Missing Metric	Proposed Approach
[11]	Longitudinal Health Impacts.	15–20% stunting drop.	HALYs for chronic diseases.	10-year cohort study, 5000 households.
[10]	Climate–Health Interactions.	2–5 °C cooling.	Malaria incidence reduction (%).	GIS-based vector modeling, tropics.
[14]	Policy–Health Integration.	10% strategies link health.	Nutrition-focused subsidy adoption.	Policy analysis across 50 countries.
[6]	Socioeconomic Determinants.	25% diet boost with tenure.	Stunting variance by tenure type.	Regression analysis, 10 regions.
[21]	Economic–Nutritional Trade-offs.	25% diversity loss.	Cost–benefit ratio (nutrition vs. profit).	Comparative trials, 5 systems.
[40]	Understanding the varying food-related experiences based on agrarian social positions (e.g., land ownership status).	Landless laborers within AFS are more vulnerable to food insecurity than peasant farmers or migrants who own land elsewhere.	Detailed comparative food security/access data based on land tenure status within specific AFS contexts.	Further research on the relationship between land access, social position, and food security for migrant/landless laborers in AFS regions. Secure land access for laborers.
[40]	How to effectively balance market demands and household provisioning needs in peasant AFS.	Peasants struggle to articulate both market production and household subsistence from AFS due to external pressures (markets, policies).	Metrics quantifying the trade-offs and potential synergies between cash crop production and subsistence farming within diverse AFS.	Revalue the non-monetary benefits of AFS and promote agroecological food production and equitable relationships through transdisciplinary collaboration involving policymakers, academics, NGOs, businesses, and civil society.
[41]	Limited understanding of how specific AFP attributes influence individual nutritional status, especially in children.	AFP diversity attributes (species richness, structural complexity) correlate with household food access and dietary diversity, particularly during food shortage seasons.	Specific linkages between consumption of diverse AFS products and individual/child anthropometric measures or micronutrient status.	Promote diversity within AFPs, focusing on helpful plant groups, including edible and storable crops needed during shortage seasons.
[42]	Lack of information on climate change impacts and adaptation strategies for marginalized indigenous communities.	Indigenous communities perceive climate variability impacts (erratic rainfall, drought) on agroforestry, leading to reduced yields, biodiversity loss, economic hardship, and dietary changes.	Quantitative data systematically links specific climatic changes to agroforestry productivity, biodiversity, and nutritional outcomes in specific indigenous contexts. Standardized methods for assessing FADI.	Support community-identified sustainable adaptation strategies (e.g., climate-resilient indigenous crops, seed saving, forest foods). Provide knowledge and technology to improve farm resilience.
[43]	Economic viability and business models for food forests, particularly for scaling up.	Most food forests perform well environmentally and socially but struggle economically. Mature sites with diverse income streams or specific high-value products/services show viability.	Comprehensive financial data, yield tracking, and standardized business performance metrics for food forests. Quantification/monetization of ecosystem services.	Develop specific training on food forest business practices. Explore cooperative ownership models (cooperatives, land trusts, foundations). Compensate for ecosystem services.
[44]	Optimizing phytochemical content in agroforestry nuts and berries through breeding and processing.	Nuts and berries from temperate AFS contain beneficial phytochemicals linked to reduced risk of CVD, hypertension, and type II diabetes.	Data on how specific breeding programs or processing techniques affect the concentration and bioavailability of key phytonutrients in AFS products.	Implement plant breeding programs focused on biofortification of health-promoting compounds. Select/develop processing techniques that preserve phytonutrients—reorient food policies to prioritize these systems.
[45]	Lack of economic/financial analysis of indigenous agroforestry models focusing on food security.	Agroforestry is a traditional indigenous practice crucial for subsistence, income, medicine, and culture. Economic studies show viability, often higher than monoculture.	Detailed economic and financial viability assessments (NPV, IRR, CBR, etc.) are specifically designed for indigenous agroforestry models with food security as a primary goal.	Conduct economic viability analyses tailored to indigenous contexts, species, and food security goals—structure AFS arrangements to provide short-, medium-, and long-term returns.
[40,45]	Understanding barriers to agroforestry adoption by farmers, including indigenous communities.	Barriers include land tenure insecurity, focus on immediate needs over long-term benefits, lack of financial resources, and cultural/ethnic factors influencing management practices.	Comparative analysis of adoption rates and influencing factors across different cultural and socioeconomic groups.	Develop public policies focused on specific community needs, including immediate returns. Address land tenure issues. Incorporate traditional knowledge and ethnic preferences in AFS design.
[45]	Gender disparities in agroforestry management and decision-making within indigenous communities.	Women are crucial for labor, food security, and income generation but often excluded from decision-making and face barriers like unequal land access.	Quantified data on women’s vs. men’s labor input, income control, and decision-making power in diverse indigenous agroforestry contexts.	Promote gender equality in AFS through targeted policies and extension services. Empower women as agents of transformation. Address land ownership inequalities.

**Table 3:** Agroforestry’s scalable contributions.

Contribution	Quantified Impact	Scientific Advance	Real-World Potential
Dual-impact quantification	0.231% food security per 1% trees	Merges agriculture and epidemiology	10% global malnutrition cut by 2040
Synergy identification	15–20% stunting, 0.5–2 Mg C/ha	Links biodiversity to health	USD 50B health savings, 1 Gt C stored
Transdisciplinary framework	15% heat death reduction	New HALYs/carbon/nutrition metric	Policy shifts in 20 nations by 2035
Documents AFS food provisioning in Chiapas peasant/migrant households; highlights conflicts between traditional and industrial food systems.	108 plant species recorded; 62.5% of families face seasonal food budget shortages; coffee AFS food species richness correlated with shade species richness (*p* < 0.05).	Integrates analysis of agrobiodiversity, food provisioning, socioeconomic pressures (markets, policy), and dietary shifts; distinct analysis of peasant vs. semi-proletarian migrant food experiences.	Informs policy by highlighting the need to value local food systems/knowledge and secure land access for laborers; guides agroecological transitions by identifying conflicts (e.g., food preference changes).
Assesses trade-offs between coffee AFS and Food and Nutrition Security (FNS) for Ethiopian smallholders, comparing different AFS types across seasons.	Species richness/stories correlated with food access security; home-garden structure/exotic species correlated with child biometrics (shortage season); combining 3 AFP types improved dietary diversity.	Quantifies links between specific AFS attributes (diversity, structure) and FNS dimensions (access, diet, child biometrics); demonstrates synergy: combining multiple AFS types enhances resilience more than single systems.	Recommends promoting diverse edible/storable crops within AFS for seasonal FNS; advises caution against over-specialization in commodity AFS; offers metrics for evaluating FNS impacts of AFS interventions.
Investigate perceived climate change impacts on AFS, diet, and diversity in an indigenous Indian community; identify mixed-method adaptation strategies.	Low agroforestry diversity (FADI = 0.21 ± 0.15); cereal-dominant diets observed; 85% HHs receive partial PDS aid; 52% HHs in debt.	Integrates community climate perceptions with quantitative AFS diversity (FADI) and diet data; develops pathway model linking climate -> AFS -> socioeconomics -> diet; documents sustainable and potentially maladaptive coping strategies.	Underscores the need for policy supporting indigenous traditional knowledge and climate-resilient crops; highlights the vulnerability of specific groups to climate impacts on food systems; points to potential conflict between hybrid promotion and biodiversity.
Compiles global evidence on food forest services and assesses their sustainability (social, environmental, economic) via literature (>200 sites) and case studies [14].	Sample (n = 209): 40% focus on education, 32% community, 11% food production. Assessed 14 sites: generally strong social/environmental scores, but 8/14 economically weak (lacked business plans).	Systematically catalogues food forest services; develops and applies a multi-criteria sustainability assessment framework for food forests; identifies standard organizational models and management issues.	Provides practical insights for food forest development (entrepreneurs, officials); identifies the need for economic viability improvements via training and business planning; suggests cooperative ownership models for scaling.
Reviews literature on health benefits (CVD, diabetes, hypertension) of nuts/berries from temperate AFS, linking AFS products to disease prevention.	Cites evidence for walnuts reducing coronary heart disease risk; cites the potential for berries in mitigating hypertension, type II diabetes, CVD.	Synthesizes evidence connecting specific temperate AFS products (nuts, berries) to diet-related disease mitigation; explicitly proposes designing AFS for health outcomes via biofortification and processing.	Offers health rationale for selecting specific species in temperate AFS design; suggests policy reorientation toward production systems with health benefits; highlights market potential for value-added processing preserving phytonutrients.
Systematically reviews AFS adoption by indigenous peoples (2010–2020), focusing on traditional practices, FNS, economic viability, and women’s roles.	Reviewed 92 works. Found AFS is often more economically viable and less risky than monoculture. Found women vital but often lack decision power/land rights.	Synthesizes evidence across multiple dimensions for indigenous AFS; confirms economic viability but notes gap in analyses tailored to indigenous FNS goals; emphasizes integral cultural/spiritual role of AFS.	Validates the importance of AFS as a traditional indigenous practice for subsistence, culture, and biodiversity; informs policies promoting AFS for FNS and poverty reduction in indigenous contexts; highlights the need to address gender inequality in AFS projects.
